# Patients’ experience with chimeric antigen receptor T-cell therapy for DLBCL in China: a qualitative study

**DOI:** 10.1007/s00520-023-07763-x

**Published:** 2023-04-26

**Authors:** Yiwen Mao, Lihong Huang, Haitao Ruan, Yue Guo, Sha Ni, Yuying Ling

**Affiliations:** 1 Department of Nursing, Tongji Hospital, Tongji Medical College, Huazhong University of Science and Technology, No.1095 Jiefang Avenue, Qiaokou District, Wuhan, 430030 China; 2 School of Nursing, Tongji Medical College, Huazhong University of Science and Technology, Wuhan, Hubei China; 3 Florence Nightingale Faculty of Nursing and Midwifery, King’s College London, Strand, London, UK

**Keywords:** CAR T-cell therapy, Diffuse large B-cell lymphoma, Experience, Qualitative study

## Abstract

**Purpose:**

The experiences of patients with diffuse large B-cell lymphoma (DLBCL) treated with chimeric antigen receptor (CAR) T-cell therapy have received little attention. This study aimed to explore the treatment experiences of patients with relapsed or refractory (R/R) B-cell lymphoma during CAR T-cell therapy in China.

**Methods:**

This descriptive qualitative study was conducted using face-to-face semi-structured interviews with 21 DLBCL patients 0–2 years after CAR-T infusion. Two researchers independently coded the interviews in MAXQDA 2022, and the original data were analyzed by conventional content analysis.

**Results:**

Four themes emerged from the transcripts: (1) physiological distress, (2) functional impacts, (3) psychological experience, and (4) support requirement. Participants expressed 29 short-term or long-term symptoms related to their disease and treatment, influencing their daily life and function in a social setting. The participants expressed different negative emotions, polarized expectations about efficacy, and over-reliance on authoritative medical care. Their major concerns and hopes were achieving life goals, being treated with respect, obtaining more information about CAR T-cell therapy, and receiving government financial sponsorship.

**Conclusions:**

The patients experienced short-term and long-term symptoms of physical distress. Patients who have experienced failure in CAR T-cell therapy also experience strong negative emotions, such as dependency and guilt. They also require authentic spiritual and financial information that is authentic. Our study may guide the development of standardized and comprehensive nursing care for R/R DLBCL patients undergoing CAR T-cell therapy in China.

## Introduction

China is facing a growing disease burden owing to lymphoma [1]. The Global Burden of Diseases, Injuries, and Risk Factors (GBD) study of 2019 showed that China accounted for 10.8% of new cases of non-Hodgkin’s lymphoma (NHL), with an increasing number of patients [2]. In September 2021, a new biological drug, the chimeric antigen receptor (CAR) T-cell product targeting CD19: KTE-X19 (Relma-cel), was officially approved for clinical use in China for relapsed or refractory large B-cell lymphoma.

CAR T-cell therapy is typically administered to patients who do not respond to first-line treatment, such as R-CHOP or rituximab-cyclophosphamide, hydroxy daunomycin, oncovin, and prednisone or autologous hematopoietic stem cell transplantation (ASCT) [3–5]. CAR T-cell therapy works by isolating and activating a patient’s T cells, through genetic modification and expands the number of T cells in vitro. The laboratory process allows CAR-T cells to be installed with the “GPS” function of targeted recognition and killing of cancer cells, followed by reinfusion into the patient [6]. Complete remissions (CRs) for relapsed or refractory (R/R) diffuse large B-cell lymphoma (DLBCL) treated with CAR T-cell therapy have been reported to be approximately 40 to 60% [7]. The advent of CAR T-cell therapies has provided new hope for R/R DLBCL patients [8].

However, compared with traditional radiotherapy and chemotherapy, CAR T-cell therapy is recognized for two unique adverse events: cytokine release syndrome (CRS) and immune effector cell-associated neurotoxicity syndrome (ICANS) [9, 10]. CRS is the most common toxic reaction, and an acute systemic inflammatory response develops in 70% of patients after CAR T-cell therapy [11, 12]. The earliest symptoms in most patients are fever, followed by tachycardia, tachypnea, hypotension, hypoxemia, and multiple organ failure [13]. ICANS usually occurs after CRS, and patients experience various neurological diseases, such as aphasia, disturbance of consciousness, cognitive impairment, seizures, and cerebral edema [14, 15]. Apart from together, CRS typically occurs within 2 weeks and ICANS usually occurs in a delayed form for 3~4 weeks after CAR T-cell infusion [9]. However, it was previously demonstrated that patients undergoing CAR T-cell therapy may experience long-term adverse reactions, such as memory disorder, disorientation, infection, or B-cell proliferation [16]. These long-term CAR T-cell therapy–related adverse effects seriously affect the patients’ quality of life. Therefore, what kind of treatment experience patients will have needs to be explored under the unique adverse events brought about by CAR T-cell therapy.

According to previous studies, patients with DLBCL undergoing radiotherapy, chemotherapy, or ASCT have physiological and psychological effects, seriously affecting their quality of life [17, 18]. Similarly, patients undergoing CAR T-cell therapy experience adverse reactions and discomfort symptoms that affect their behavior, ability, mood, etc. [19]. Furthermore, patients experience different symptomatic, functional, and psychological effects in the acute phase (0–3 months) and long-term (more than 3 months) of CAR T-cell therapy [20]. Currently, there is limited literature on the long-term influence of CAR T-cell therapy, especially for more than 1 year. Therefore, we selected patients with CAR-T infusion 0–2 years prior as interviewees, hoping to explore the treatment experience more comprehensively. Understanding the treatment experience of cancer patients could provide better and more targeted nursing. Better care can help improve patients’ quality of life [21], enhance the relationship between doctors and patients, and possibly modify treatment-related decisions [22].

This qualitative study aimed to explore the physical discomfort experienced by patients during CAR T-cell therapy. The second aim of this study was to differentiate the experiences of R/R DLBCL patients who underwent another treatment previously from those who underwent CAR T-cell therapy, as well as the kind of support these patients needed.

## Methods

Descriptive qualitative research methods were used in this qualitative study [23]. Qualitative procedures were led by the first two authors (Mao YW and Ruan HT), who have extensive experience in qualitative studies, including interviewing patients and data analysis. Semi-structured and face-to-face interviews were conducted to explore patients’ treatment experience with R/R DLBCL after CAR T-cell therapy [24].

### Participants

The study was conducted at a public hospital in Wuhan, Hubei, China, and interviews were conducted in the inpatient Hematology Department. The purposive sampling method was used to select participants with diverse CAR-T infusion types, ages, times since CAR-T infusion, and different levels of adverse reactions to ensure maximum variation between patients. The patients received commercial CAR-T products from two Chinese companies (FOSUNKite and JW Therapeutics): axicabtagene ciloleucel and relmacabtagene autoleucel. To ensure a comprehensive treatment experience for patients receiving CAR T-cell therapy, we also selected patients with different CAR-T product access channels: commercial or clinical trials. The inclusion criteria were patients with relapsed or refractory B-cell lymphoma, aged 18 years or older, who had CAR-T infusion 0–2 years prior and could communicate in Mandarin. Participants were excluded if they had other significant illnesses, hearing impairments, or psychiatric abnormalities.

### Procedures and data collection

Interviews were conducted based on the patients’ free time and comfort. The interviews were conducted in a single room in the inpatient department or a spare clinic in the outpatient department.

The final interview script (Table 1) was developed through expert group discussions and was pre-tested by two participants. The expert group consisted of clinical hematology professors, head nurses, and sociology and psychology experts with more than 10 years of experience.

**Table 1:**
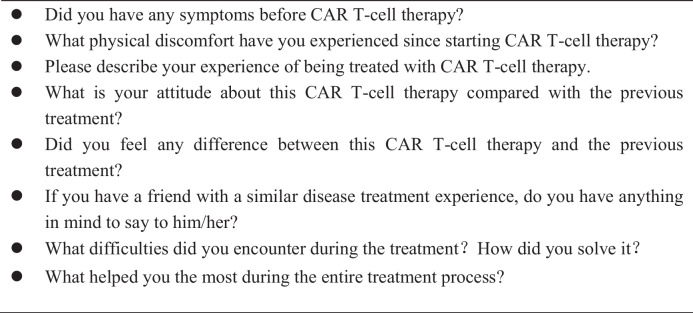
Interview guide. CAR-T, chimeric antigen receptor T-cell

After the pre-interview with the two participants, the initial interview outline was slightly revised, and these two cases were excluded. All team members of the expert group agreed to the final script questions. The interviews began with an open-ended question such as “How are you feeling these two days?” Patients were allowed to express themselves freely without interruption. Based on the patient’s answers, the researcher asked additional questions as deemed appropriate to better guide patients in describing their CAR T-cell therapy experience. The interviews lasted between 42 and 65 min.

According to the data collection guidelines for qualitative research, data were collected until no new information or new themes emerged [25]. Therefore, 21 lymphoma patients were interviewed, and the data were saturated without new information emerging after the 19th interview. Sociodemographic and clinical data were additionally derived from the participants and electronic medical records.

### Data analysis

The traditional content analysis method described the participants’ experiences with CAR T-cell therapy [26]. Two experienced qualitative researchers transcribed the recordings verbatim into text for less than 24h after the interview. The transcribed text was then given to the patient for confirmation after double-checking to ensure the accuracy of the transcription.

Qualitative software MAXQDA version 2022 was used for the encoding process. Firstly, the first and second authors carefully read the entire transcript to gain a sense of the complete data and ensure data immersion. Discrepancies were resolved through discussions. The transcribed text was read line by line, followed by categorization of the information for open coding of statements related to the patient’s physical and psychological experiences with the CAR T-cell therapy. Actual words coded as similar and relevant to physical discomfort, negative emotions, and their coping methods were categorized into themes and sub-themes. Common associations between themes were clustered and corresponding data examples were extracted. Finally, the text was cycled through until no new themes or subthemes were presented. The final theme was identified using descriptive exploratory analysis.

## Results

### Descriptive results

A total of 21 patients with R/R DLBCL patients participated in the interviews. The demographic and clinical characteristics of the participants are presented in Table 2. The participants included 15 male patients. Nine patients were treated with commercial CAR T-cell therapy, and 12 underwent clinical trials involving CAR T-cell therapy. Six participants were interviewed within 1 month of their CAR T-cell infusion, whereby their vital signs were stable, and the patients were comfortable, while four patients each were interviewed between 1–3 months, 3–6 months, and 1–2 years following their CAR T-cell infusion (patients came to the inpatient unit or outpatient clinic for review), and three patients were interviewed between 6 and 12 months following their infusion.

**Table 2:** Demographic and partial clinical characteristics (*n* = 21)

	Mean	SD
Age	45.6	15.8
	Frequency percentage	Frequency percentage
Sex		
Male	15	71.4
Female	6	28.6
Nationality		
Han	20	95.2
Ethnic minorities	1	4.8
CAR-T type		
Commercial	9	42.9
Clinical trial	12	57.1
Time since CAR-T infusion		
0~1 months	6	28.6
1~3 months	4	19.0
3~6 months	4	19.0
6~12 months	3	14.3
1~2 years	4	19.0
Toxicity		
Neither CRS nor neurotoxicity	4	19.0
CRS		
1 level	6	28.6
2 level	5	23.8
3 level	5	23.8
4 level	1	4.8
ICANS		
1 level	1	4.8
2 level	1	4.8
Employment status		
Unemployed	5	23.8
Retired	4	19.0
Medical leave of absence	7	33.3
Employed outside the home	5	23.8
Medical insurance type		
Medical insurance	15	71.1
Commercial insurance	3	14.3
Rural medical insurance	3	14.3
Education		
Junior high school graduate	6	28.6
High school graduate	5	23.8
College graduate	7	33.3
Graduate degree	3	14.3

*CAR-T*, chimeric antigen receptor T-cell; *CRS*, cytokine release syndrome; *ICANS*, immune effector cell-associated neurotoxicity syndrome; *SD*, standard deviation

### Qualitative results

**Fig. 1:**
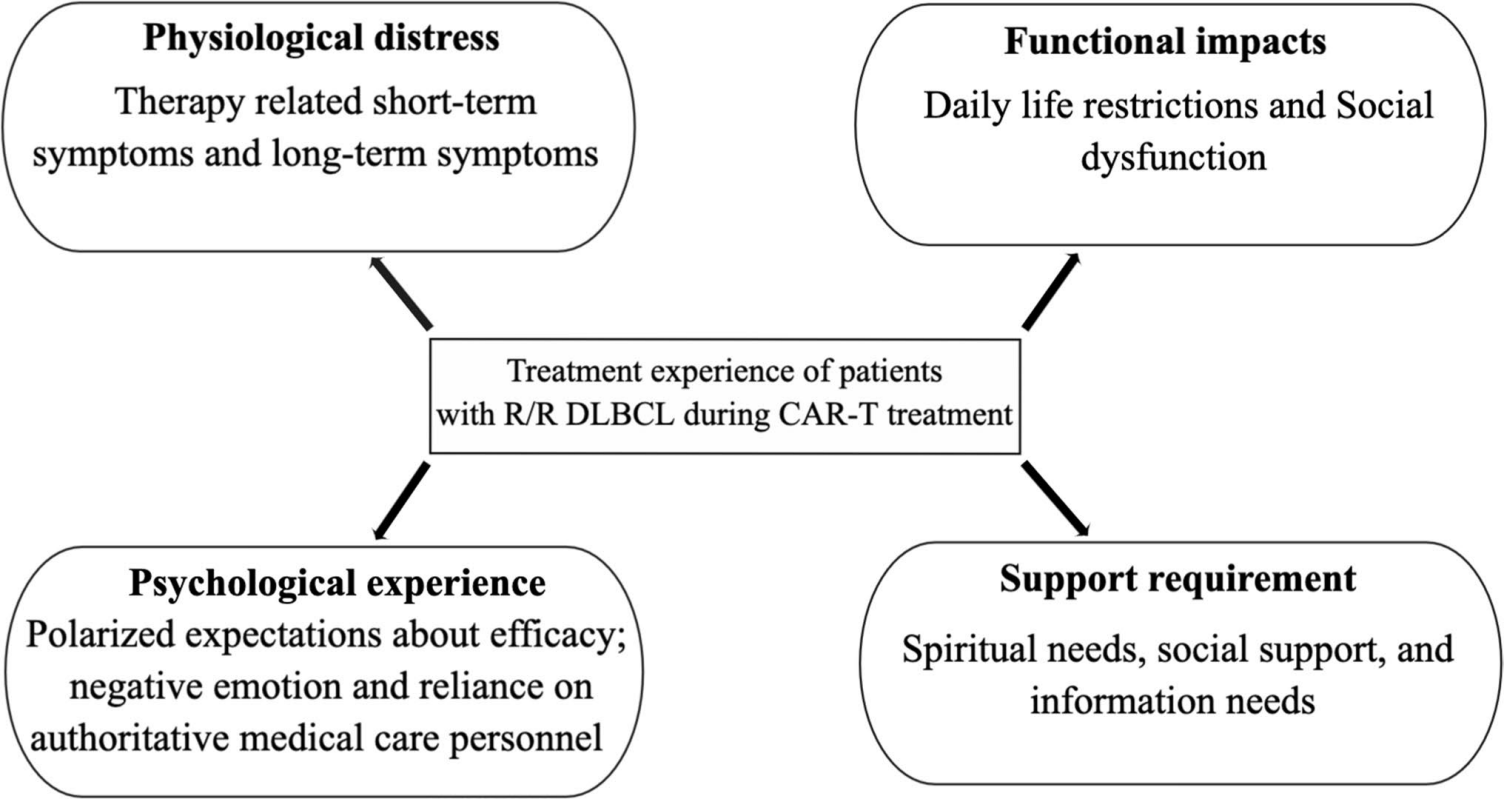
Themes and subthemes of treatment experienced by patients with R/R DLBCL during CAR T-cell treatment

Four themes related to patients’ physical discomfort, negative emotions, and their requirements of CAR T-cell therapy were derived from the semi-structured interview transcripts through qualitative thematic content analysis: (1) physiological distress, (2) functional impact, (3) psychological experience, and (4) support requirements (Fig. 1). Table 3 lists these themes.

**Table 3:** Relevant quotations for each sub-theme

Themes	Sub-themes	Quotations
Physiological distress	Therapy-related short-term symptoms	Loss of appetite “My appetite is not very good. I don't want to eat very much. Now I don't have a fever, but my appetite is still not very good.” (Participant No.1)
		Excessive sweating “Mainly the neck and chest area, I sweat a little too much, also like to drink water.” (Participant No.1)
		Pain“Because I have lymphoma and the tumor is huge, I have pain, which is slightly relieved by the painkillers. After the CAR T-cell therapy, the other response is OK, that is, my tumor compressed the nerve, so I have back pain.”(Participant No.6)
		Vomit “After the CAR T-cell infusion, I had a poor appetite and vomited for several days.” (Participant No.1)
		Discomfort at tumor location “I have no pain in my body. When I did CAR-T treatment, I felt pain in the lump and was uncomfortable. Then I couldn't sleep at night because of the pain, and it would be better to sit up.”(Participant No.14) “A few days after the CAR T-cell infusion, I sometimes felt something wriggling around here (in the mass), something twitching.” (Participant No.16)
		Dry mouth “My mouth feels dry and I can't even eat. I can only eat something with soup.”(Participant No.7)
	Therapy-related long-term symptoms	Fatigue “I'm not as strong as I used to be, but I'm OK. I usually walk in the yard, not to do those very strenuous exercises, just take a walk, about 2 to 3000 steps a day.” (Participant No.3)
		Memory loss “There's a little bit of a difference in memory. Now my reaction is slow, my memory is not good, and sometimes I even forget whether I have eaten (Laugh).” (Participant No.12)
		Rash “When I did CAR T-cell therapy, I became swollen all over my body, swollen hands, eyes, itching all over my body, which is like a skin disease, and I have a very bad rash, and I still have a rash and occasionally itch for a year now.” (Participant No.7)
		Orientation disorder “I feel very confused, yesterday I went to have a PET examination, I can’t find my way, but I used to know the direction, and then I had to call my wife and ask her to take me for a check.” (Participant No.8)
Functional impacts	Daily life restrictions	“Now I'm a little slower than I used to be, and when I come home from the hospital, I always miss my reflexes when I'm driving, and then I hit another car.” (Participant No.8)
		“I can't walk a long distance and I'm tired if I stand for a long time. I'm not as energetic as before.” (Participant No.9)
		“Because my platelet is very low, it had become more troubling, and I usually have to be careful to avoid collision, and I cannot walk too long. I must be cautious and gentle when brushing my teeth. Otherwise, the gumboils easily bleed.” (Participant No.13)
	Social dysfunction	“I had to inject PD-1 every month, and it was very inconvenient for me to return to work. I did not know how to ask for leave from my boss, because every time I was admitted to the hospital for injection, it took 4 to 5 days.” (Participant No.17)
		After I got sick, my friends intentionally stayed away from me and didn't actively deal with me anymore. (Participant No.17)
Psychological experience	Polarized expectations about efficacy	“This is my second relapse, and since my first relapse I've had lower expectations for everything, and the lower my expectations are, the less frustrated I was if something doesn't work out.” (Participant No.12)
		“This time, I came for CAR T-cell therapy with great hope. Because I have suffered a lot from chemotherapy, so I am very confident and interested in doing CAR T-cell therapy, and I hope I can achieve complete remission this time.” (Participant No.15)
	Negative emotion	“I feel that I am a burden to my family because of my illness. I used to take care of my parents, but now they have to take care of me. Moreover, I have no energy to take care of my children.” (Participant No.21)
		“Before my treatment, I had some ideas and plans for many things. I felt that I would face a big test and there might be an accident.” (Participant No.16)
		“Because after the chemotherapy, I had lost my hair and rarely went out because sometimes acquaintances would ask, and I now pay attention to personal privacy. Because I was young and didn't want my child to grow up and be told that his father had something wrong, even my wife didn't know what was wrong with me.” (Participant No.19)
	Reliance on authoritative medical care personnel	“Professor Zhou is a spiritual pillar for patients like us because he represents our hope to live.” (Participant No.16)
		“CAR T-cell therapy was my only hope of survival, and other treatments had no effect”(Participant No.10)
		“I only listen to doctors, and I trust you (the medical staff) and no one else.”(Participant No.4)
Support requirement	Spiritual needs	“I often read books and feel that books are my spiritual ballast. Every time I encounter trouble, I will read a book and my mood will be much better.” (Participant No.12)
		“I really want to get better soon, and then I can do the things I like, such as travel to a different place, which is my dream.” (Participant No.8)
		“I don't like people asking me about my illness as soon as they see me. I need to be seen as a normal person.” (Participant No.21)
	Information need	“I need to know more about CAR T-cell therapy, such as the response rate of CAR T-cell therapy, whether there is a possibility of relapse after treatment, any serious adverse effects, and other more effective treatments. The more I know, the more I feel at ease.” (Participant No.6)
	Social support	“The cost of CAR T-cell therapy is too high for the average family to afford. Because I am sick and can't go to work, my quality of life has declined significantly. I hope the government can give me some subsidies or cover part of the cost of CAR T-cell therapy as medical insurance.” (Participant No.15)
		“I have been ill for more than two years. I have spent much money, and I do not know how much money I will spend in the future. I have added much financial burden to my family” (Participant No.11)
		“Because I am an ethnic minority, I have a special dietary requirement, such as not eating pork. Due to the impact of COVID-19, I had to order food when I was hospitalized, as I was not used to eating food delivered by the hospital.” (Participant No.17)

## Theme 1: physiological distress

**Fig. 2:**
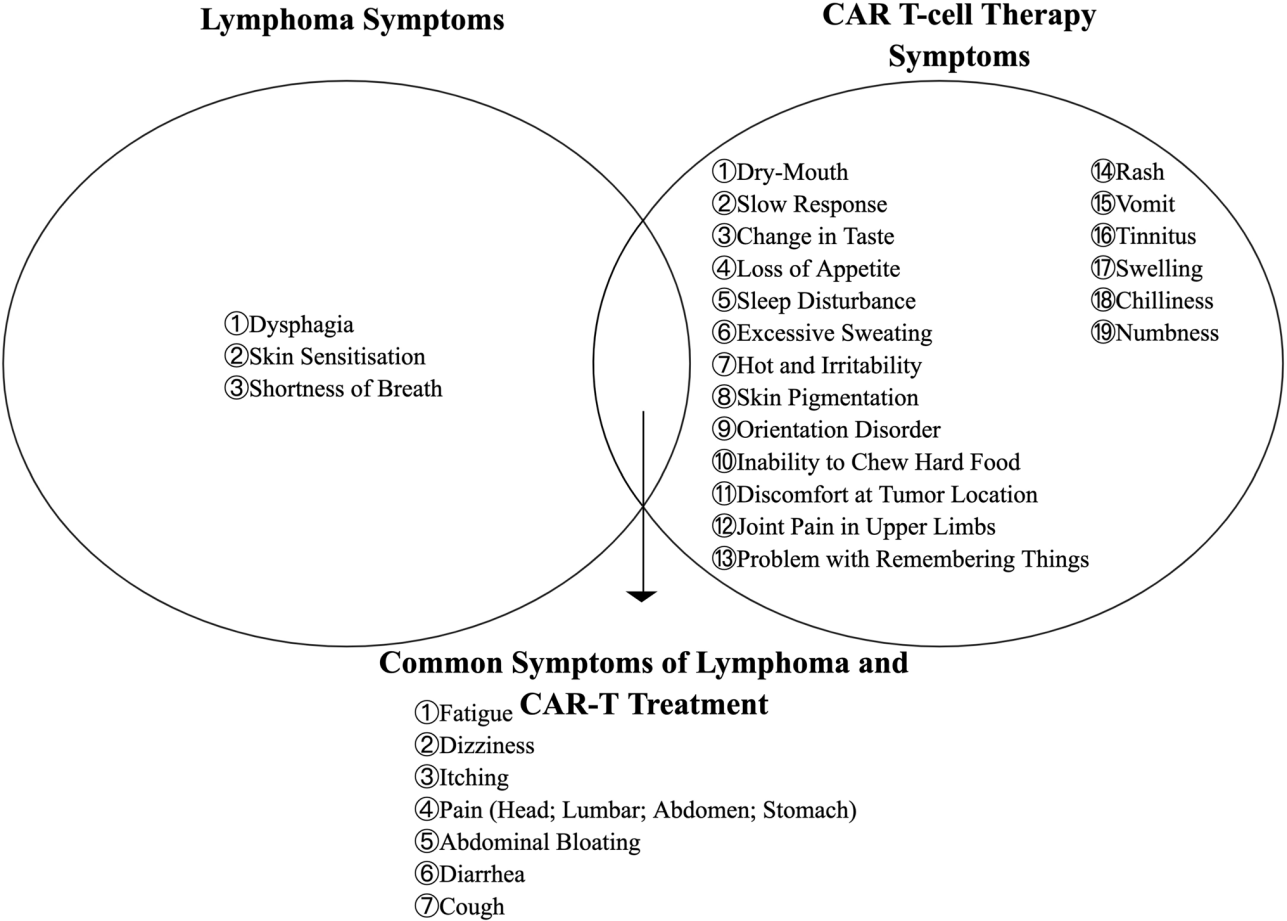
Symptoms expressed by patients who had received CAR T-cell therapy

All participants reported experiencing diverse physical discomfort following CAR T-cell therapy. To briefly distinguish between symptoms of lymphoma and symptoms after CAR T-cell therapy, patients were interviewed to recall what physical discomfort was experienced before CAR T-cell treatment, what kind of new symptoms appeared after CAR T-cell therapy, and whether their previous symptoms had improved. The symptoms identified in patients who had received CAR T-cell therapy are revealed in Fig. 2.

### CAR T-cell therapy symptoms

In this qualitative interview, patients with DLBCL expressed 24 types of physiological discomfort after CAR T-cell therapy. These patients had been treated with other treatments such as chemotherapy and were expected to be concerned with symptoms related to CAR T-cell therapy and compared with symptoms with previous therapy.

Fatigue, loss of appetite, excessive sweating, pain, vomiting, dizziness, discomfort at the tumor location (feeling itchy, sore, or twitchy), and dry mouth were the most common symptoms reported by the patients. Except for fatigue, most of these symptoms can be spontaneously relieved with rest; however, the time to recovery may differ between patients. The prolonged symptoms identified by patients during CAR T-cell therapy in the qualitative interviews included fatigue, memory loss, rash, and disorientation, which affected their daily life long-term.

## Theme 2: functional impacts

### Daily life restriction

The patients in the qualitative interviews described how symptoms after CAR T-cell therapy affected their daily lives. For example, the help of family members was needed when taking a bath, while brushing their teeth became a challenge owing to low platelet counts. Furthermore, patients with disorientation could not leave their homes alone, walk or stand for prolonged periods soon after CAR T-cell infusion, and could only eat heated and sterilized food because of prolonged reduced immunity.

### Social dysfunction

Patients also stated that they needed more than monthly programmed cell death protein-1 (PD-1) therapy injections. The regular hospital visits associated with the treatment affected their employment and job hunt opportunities. Many patients reported that their interactions with relatives and friends had decreased since they became ill, whereas their social interactions had completely stopped. Most patients reported that although their daily lives were affected, the disturbances were acceptable. A few patients expressed high hope of fully returning to a normal life after receiving CAR T-cell therapy.

## Theme 3: psychological experience

### Polarized expectations about efficacy

Nearly all participants reported a change in their attitude toward the efficacy of CAR T-cell immunotherapy due to relapse or treatment failure. Their expectations were polarized when confronted with “new hope” again. During the interview, the patients responded that they actively lowered their expectations for any matter after the recurrence of their disease due to fear of disappointment.

Some participants initially expressed having high expectations but gradually decreased after CAR T-cell therapy. The physical discomfort and difficulties caused by the treatment made them look at CAR T-cell therapy more objectively. However, some patients have contrasting opinions. They regarded CAR T-cell therapy as the last hope and had reasonable expectations for the treatment effect.

### Reliance on authoritative medical care personnel

Many patients reported dependence on authoritative personnel from medical institutions for information regarding cutting-edge CAR T-cell therapy technology. Speaking to their attending professors, even on topics unrelated to their disease, comforted the patients. In the event that their professor died, some patients became temporarily flustered, temporarily. Many patients also expressed a fear of CAR T-cell immunity.

### Negative emotion

Long-term treatment, high medical costs, and worsening physical discomfort have led to nearly all patients experiencing negative emotions related to the therapy. Patients expressed destructive emotions using negative words, such as guilt, anxiety, worry, irritability, and passive behavior.

## Theme 4: support requirement

### Spiritual needs

Seven patients indicated they wanted spiritual-based support. Some patients regarded books, music, dolls, diaries, and other items as their spiritual sustainment and relieved their negative emotions through “inner” dialogues. Most participants expressed a need to protect their dignity and hoped they would not be treated differently or casually discussed because of their illness. Four participants felt that they needed help to achieve their life goals after being rendered weaker by illness and needed help to realize their value and become enriched.

### Information needs

In addition to spiritual needs, most patients also expressed their information and material needs. Nine respondents shared that because CAR products were only recently approved for clinical use in China, they wanted to know more about CAR T-cell technology and their disease to resolve their doubts.

### Social support

Almost every patient mentioned that their quality of life was severely reduced due to the high cost of CAR T-cell therapy and the lack of funds. In particular, patients treated with commercial CAR T-cell therapy reported a greater financial burden as well as an urgent desire to return to work. Both expressed a desire to receive government funding for their CAR T-cell therapy.

In addition, a few patients expressed the hope of receiving supportive medical care during hospitalization, such as diets suitable for certain minorities and a choice of infusion time.

## Discussion

This qualitative study explored the treatment experience of R/R DLBCL patients during CAR T-cell therapy in China. R/R DLBCL patients’ experiences with CAR T-cell therapy were related to physiological distress, psychological experience, functional impact, and support requirements, similar to other qualitative studies [19, 27]. However, CAR T-cell therapy symptoms, daily life impact, social dysfunction, reliance on authoritative medical care personnel, and spiritual and material needs were newly emerging subthemes identified through this qualitative study. These emerging subthemes reflect Chinese DLBCL patients’ experience of having “new hope” after having experienced “setbacks”.

This is the first qualitative study to explore the physical discomfort experienced by lymphoma patients during the first 2 years after CAR T-cell therapy. Patients with R/R DLBCL reported 29 uncomfortable symptoms related to CAR T-cell therapy and lymphoma. Among the commonly reported symptoms (fatigue, loss of appetite, excessive sweating, pain, vomiting, dizziness, and discomfort at the tumor location) expressed by patients, except for fatigue symptoms, the other commonly reported symptoms were short-term and developed gradually after CAR T-cell infusion but resolved spontaneously over some time. A similar finding was also reported in a previous study: symptom burden peaked approximately 2 weeks after CAR T-cell infusion and returned to normal after 60–90 days, which was related to the timing of the release of inflammatory factors [28, 29]. Fatigue, memory loss, rash, and disorientation have been reported as long-term symptoms. Patients still reported these symptoms 1–2 years after CAR T-cell infusion, seriously affecting their daily lives. We found that affected by physical discomfort, several patients reported daily life restrictions and social dysfunction after CAR T-cell therapy, such as walking ability, daily activities, and socialization, which was similar to those of Whisenant et al. (2021) [19]. It is worth focusing on the fact that it takes longer for patients to recover from functional effects due to long-term adverse reactions and symptoms. The onset of long-term symptoms may be associated with ICANS, which are common adverse effects of CAR T-cell therapy, potentially affecting patients for 1–5 years [30]. The presence of short-/long-term symptoms implies the need to collect longitudinal clinical information. Previous studies have demonstrated that early detection and intervention of CRS and ICANS can effectively reduce toxic side effects [14]. Therefore, especially during the acute phase of CAR T-cell therapy, we should focus on the onset and regression of symptoms in patients. In addition, for patients with long-term symptoms, methods to relieve their symptoms and reduce the impact of symptoms on their normal life can be studied later. Specific symptom assessment tools that can be used after CAR T-cell infusion and the frequency at which healthcare professionals should measure patients need to be further investigated.

The present qualitative study revealed that patients had rich and varied psychological experiences during their entire treatment process, such as polarized expectations about efficacy, negative emotions, and reliance on authoritative medical care personnel. Patients with disease recurrence or treatment failure have a polarized attitude toward “new hope.” One explanation is that this may be related to whether patients had a high level of dispositional optimism [31]. Patients with a high level of dispositional optimism are more likely to have a positive attitude toward facing difficulties and may have active coping strategies [31, 32]. Another explanation is that patients with cancer may experience different post-traumatic stress disorder (PTSD) and post-traumatic growth (PTG) [33]. PTSD and PTG coexist in these patients, but with different pre-trauma experiences and further growth after cancer, resulting in different types and levels of PTG after CAR T-cell therapy [34]. Some patients also learned never to give up from their previous experiences, while others grieved and could not face the rest of their treatment with a positive attitude. This suggests that for patients with R/R DLBCL whose treatment experiences were bumpier than those of other cancer patients [35], effective interventions to reduce post-traumatic stress should be emphasized. For example, mindfulness therapy combined with information support could be of great help to encourage PTG [36].

In addition, we found that patients expressed various negative emotions during the interviews, each reporting guilt and most expressing nervousness, anxiety, and fear of disease recurrence, which was in line with a previous study [37]. A possible reason for these negative thoughts is that traditional Chinese Confucian filial piety culture emphasizes filial piety to parents known by most patients [38]. DLBCL patients in our study were primarily young and middle-aged (mean age=45.6), with elders and children to take care of but were unable to because of their disease. A lack of social/family functions aggravates their negative emotions. In another context, most patients strongly depend on an authoritative medical team figure and the cutting-edge technology of CAR T-cell immune therapy. They often report treating the authoritative medical team as the “spiritual pillar” of new treatments. Currently, there is no relevant research, and for patients with treatment failure or ineffectiveness, dependence should be further explored. For instance, is the development of strong dependence related to the patient’s treatment experience and new treatment methods? Is a strong dependence good or bad? How should healthcare providers intervene? However, we can clarify that these patients have a high demand for humanistic care.

Almost every patient expressed their special needs during the CAR T-cell therapy, similar to another study [39]. The patient’s needs can be divided into three categories: spiritual needs, social support, and information needs. Most patients had high spiritual needs, especially the need to pursue and achieve meaningful life goals, which is consistent with the results of previous research [40]. The patients wanted to be respected and to have spiritual ballast. This can be explained by the recent launch of CAR T-cell therapy in China, causing the physical and therapeutic status of patients to receive more attention than spiritual needs [41]. By contrast, spiritual needs are more important for patients who have experienced failed or ineffective treatment. The experience of living in the shadow of death for a long time can prompt self-reflection and a search for meaning and purpose in life [42]. These outlooks promote health and quality of life [43], as well as alleviate their negative emotions [44, 45]. Medical staff should improve their ability to assess, care for and respond to patients’ spiritual needs, and actively support personalized spiritual needs [46]. Effective assessment tools and procedures regarding the spiritual needs of patients should be established and integrated into the nursing process to aid medical staff in completely caring for these patients [47].

In addition, this qualitative study also found that patients had a high need for social support, such as financial support and the requirement of ethnic minorities. Financial support was an essential need for each patient interviewed, consistent with a previous study [48]. The cost of follow-up treatment, toxicity management, and follow-up examinations has gradually increased the economic burden on patients receiving CAR T-cell therapy [49]. In particular, for patients receiving commercial CAR T-cell therapy, economic toxicity should be considered. Currently, China has not included the cost of CAR T-cell therapy in its medical insurance claims, and patients have to pay most of the cost out of their own pockets. More importantly, the treatment costs are high, far exceeding the average annual income level of Chinese people, and patients with hematological malignancies are severely affected by financial toxicity [48]. Therefore, the Chinese government should expand medical insurance coverage related to cancer patients’ treatment soonest and subsidize the costs associated with CAR T-cell therapy [50].

Most patients in the current study also expressed inadequate information about CAR T-cell therapy. They expressed an intense need for accurate information on remission rate, long-term survival time, and the latest research and development on CAR T-cell therapy. Unlike a previous study, patients undergoing CAR T-cell therapy and their families had a greater need to know the truth about the treatment rather than the degree of concealment to appease the patient [51]. Again, this finding may be associated with the recent availability of CAR T-cell therapy in China, causing patients to want to know more about authentic information. Another possible explanation is that CAR T-cell therapy is costly, often costing the family savings. As such, consideration of the “value for money” and whether the treatment is worthwhile requires comparing information and data. This finding reveals the importance of making precise information about CAR T-cell therapy easily available to patients or communicating this information well. In addition, organizing peer-sharing sessions with current patients undergoing CAR T-cell therapy can become a trusted space for new patients [18].

## Strengths and limitations

This qualitative study provides insights into the treatment experience of patients with R/R DLBCL patients undergoing CAR T-cell therapy. However, there are several limitations to this study. First, although the widest diversity in the sample group was used during respondent selection, the study was conducted at only one hospital in China. Thus, the results represent only a few DLBCL patients across China. Second, patients who reported high ICANS or CRS adverse reaction ratings were excluded due to poor health or died before they could be interviewed, leading to the opinion and experience of this group of patients being excluded from consideration. Third, we did not consider the impact of CAR-T product brands on the treatment experience of patients, especially in the field of physiological distress. In the future, when we explore the symptoms reported by patients after CAR T-cell therapy, we will consider this important influencing factor and further explore it.

## Conclusion

The outcome of this study provided information about the treatment experience of R/R DLBCL patients within 2 years of CAR T-cell therapy. In physiological distress, we found several long-term and commonly reported short-term symptoms. Patients who have experienced treatment failure have polarized expectations for the newly authorized CAR T-cell therapy and can become overly dependent on an authoritative medical team, as well as develop negative feelings of guilt. In addition, the sampled patients expressed a spiritual need for respect and self-fulfillment as well as a material need for more authentic information about treatment and funding. In conclusion, healthcare professionals should closely evaluate the development and resolution of short-term symptoms after CAR T-cell infusion and pay attention to the impact of long-term symptoms on patients to provide effective interventions. More attention should be paid to the patients’ negative emotions and spiritual needs to provide the effective and necessary support. The government should also provide more funding to support patients undergoing CAR T-cell therapy.
